# Spatial variations and long-term trends of potential evaporation in Canada

**DOI:** 10.1038/s41598-020-78994-9

**Published:** 2020-12-16

**Authors:** Zhaoqin Li, Shusen Wang, Junhua Li

**Affiliations:** Canada Centre for Remote Sensing, Natural Resources Canada, 560 Rochester Street, Ottawa, ON K1A 0E4 Canada

**Keywords:** Climate change, Hydrology

## Abstract

Assessing the status and trend of potential evaporation (PE) is essential for investigating the climate change impact on the terrestrial water cycle. Despite recent advances, evaluating climate change impacts on PE using pan evaporation (E_pan_) data in cold regions is hindered by the unavailability of E_pan_ measurements in cold seasons due to the freezing of water and sparse spatial distribution of sites. This study generated long-term PE datasets in Canada for 1979–2016 by integrating the dynamic evolutions of water–ice–snow processes into estimation in the Ecological Assimilation of Land and Climate Observations (EALCO) model. The datasets were compared with E_pan_ before the spatial variations and trends were analyzed. Results show that EALCO PE and E_pan_ measurements demonstrate similar seasonal variations and trends in warm seasons in most areas. Annual PE in Canada varied from 100 mm in the Northern Arctic to approximately 1000 mm in southern Canadian Prairies, southern Ontario, and East Coast, with about 600 mm for the entire landmass. Annual PE shows an increasing trend at a rate of 1.5–4 mm/year in the Northern Arctic, East, and West Canada. The increase is primarily associated with the elevated air temperature and downward longwave and shortwave radiation, with some regions contributed by augmented wind speed. The increase of annual PE is mainly attributed to the augmentation of PE in warm seasons.

## Introduction

Potential evaporation (PE) defines the maximum water loss to the atmosphere from a surface with an unlimited or non-limiting supply of available water, and the surface resistance to the flow of vapor is negligible^1–3^. PE is used as a measure of atmospheric evaporative demand (AED) that connects climate with the terrestrial water cycle and the biosphere via controlling actual evapotranspiration^4–6^. Assessing the status and trend of PE is thus essential for predicting changes in the terrestrial water cycle^4^ and the biosphere^5,6^ in future climate scenarios.

Contrasting trends of PE have been identified using Pan evaporation (E_pan_) data among regions during different periods and the tendencies are attributed to the changes in a variety of climate variables. Despite global warming, a decreasing trend of PE has been widely reported^4,7–10^. The decrease is attributed to the dimmed solar radiation^4,11,12^, the stilling of near-surface wind^9,13^, or both^14^. In contrast, an increasing trend of PE has been described as a response to global warming^15–17^ or increased solar radiation^18^. The inconsistent trends and climate drivers of changing PE are partially accounted for by sparse spatial distribution of E_pan_ measurements and scarcity of high-quality PE data, especially at large temporal and spatial scales^18^. A long-term PE dataset is particularly in demand in cold regions where climate warming is more intense than lower latitudes^19,20^.

Generating a high-quality PE dataset in cold regions faces two main challenges. Firstly, PE by E_pan_ measurements in weather stations is not available in winter when water freezes^21^. Secondly, the dynamic evolution of surface conditions of water, ice, and snow complicates the PE processes and estimation.

Evaporation models have an advantage over the evaporation pan on PE estimation in terms of data temporal continuity and spatial repetitiveness. Physically-based evaporation models are developed based on the energy available for water vaporization or the aerodynamic principle controlling water vapor transportation processes, or both^22^. Among the models, the Penman equation^1^ and the modified Penman equation^23^ that integrate both radiative energy and aerodynamic components typically outperform the single-component models^24–26^. However, there is no universal consent that any given model is consistently suitable in a specific climate^27^. How the Penman equation performs in cold regions needs to be assessed. The water freezing and thaw, snow accumulation and melt, and the dynamic change of snow albedo are essential processes in determining PE in cold regions. However, these snow and ice processes affecting PE have rarely been incorporated into estimation in previous studies.

**Figure 1:**
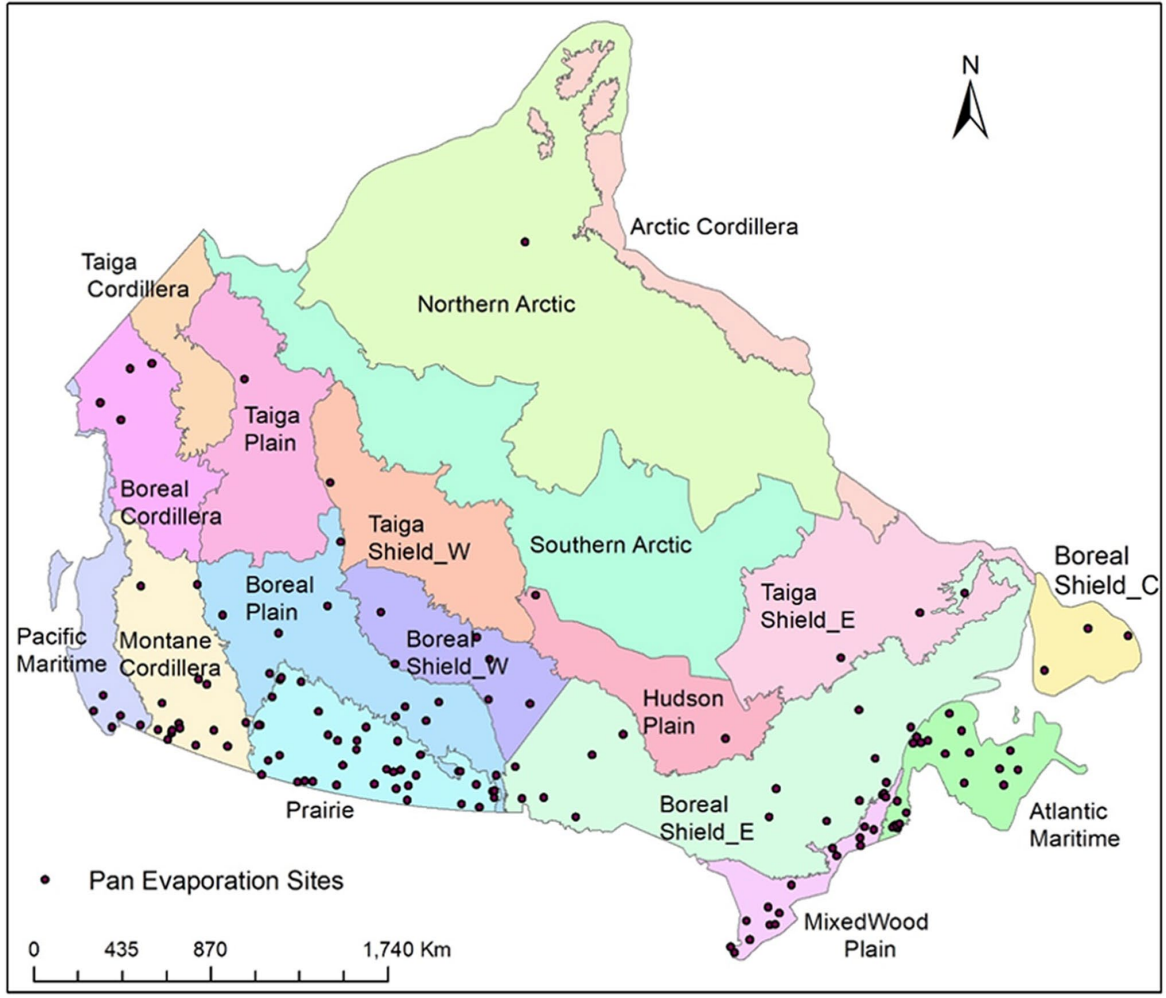
The spatial distribution of the 18 ecozones and the 141 Pan evaporation (E_pan_) sites across Canada.

In this study, the effects of snow and ice processes were integrated into PE estimation using the Ecological Assimilation of Land and Climate Observations (EALCO) model. EALCO is a land surface model that includes dynamic surface evolutions of water, ice, and snow^28,29^. The model was used to generate a daily PE dataset for 38 years (1979–2016) over entire Canada's landmass at a 10 km spatial resolution to achieve the three objectives: (1) to fill the PE data gap in cold seasons; (2) to determine the relationships between modeled PE (EALCO PE) and E_pan_ across different climate regions; and (3) to analyze the spatial distribution, seasonal variations, and long-term trends of PE. Initially, there are 15 ecozones (http://ecozones.ca/english/zone/index.html) over the Canadian landmass. We split the Boreal Shield ecozone into Boreal Shield West, Boreal Shield East, and Boreal Shield Coast, and separate the Taiga Shield into Taiga Shield East and Taiga Shield West to better recognize the spatial variations in climate (Fig. 1). Climate among 18 ecozones varies dramatically from cold and dry Arctic, semiarid Prairies, mountain climate, to humid maritime climate. Detailed climate variations among ecozones are shown in Fig. 4b–e. The EALCO PE was compared with E_pan_ measured from May to September 1979 to 2007 in 15 out of 18 ecozones, based on the availability of E_pan_ data (Fig. 1). The spatiotemporal variations and trends of PE were then analyzed.

## Results

### Comparisons of EALCO PE and E_pan_

**Figure 2:**
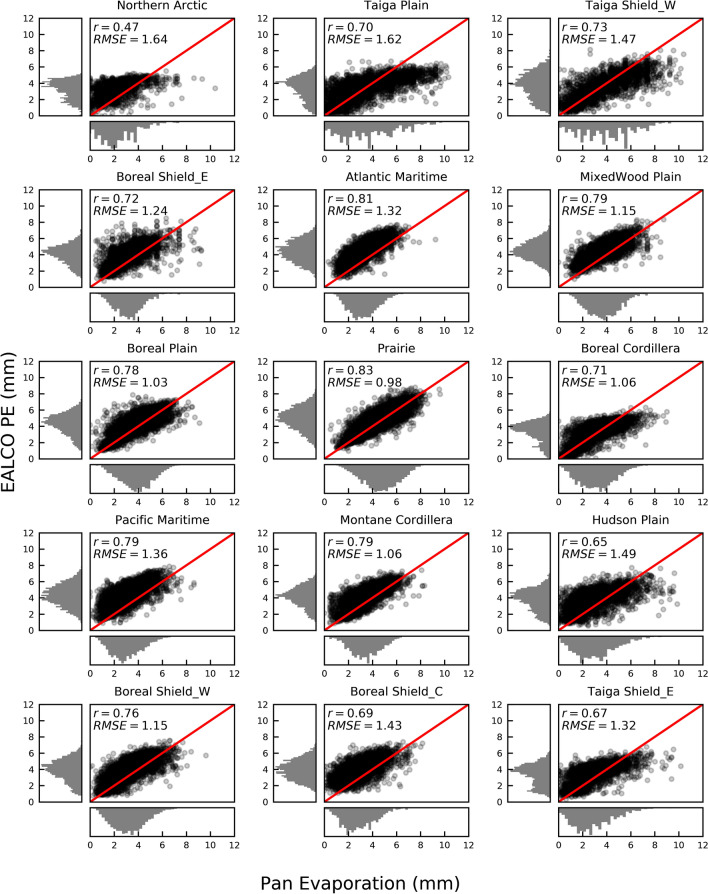
The scatterplots and the histograms of daily EALCO PE (mm) and Pan evaporation (E_pan_, mm) with the correlation coefficient (r), Root Mean Squared Error (RMSE), and 1:1 line in red in May (or June in Northern Arctic) to September 1979–2007 in 15 ecozones of Canada.

**Figure 3:**
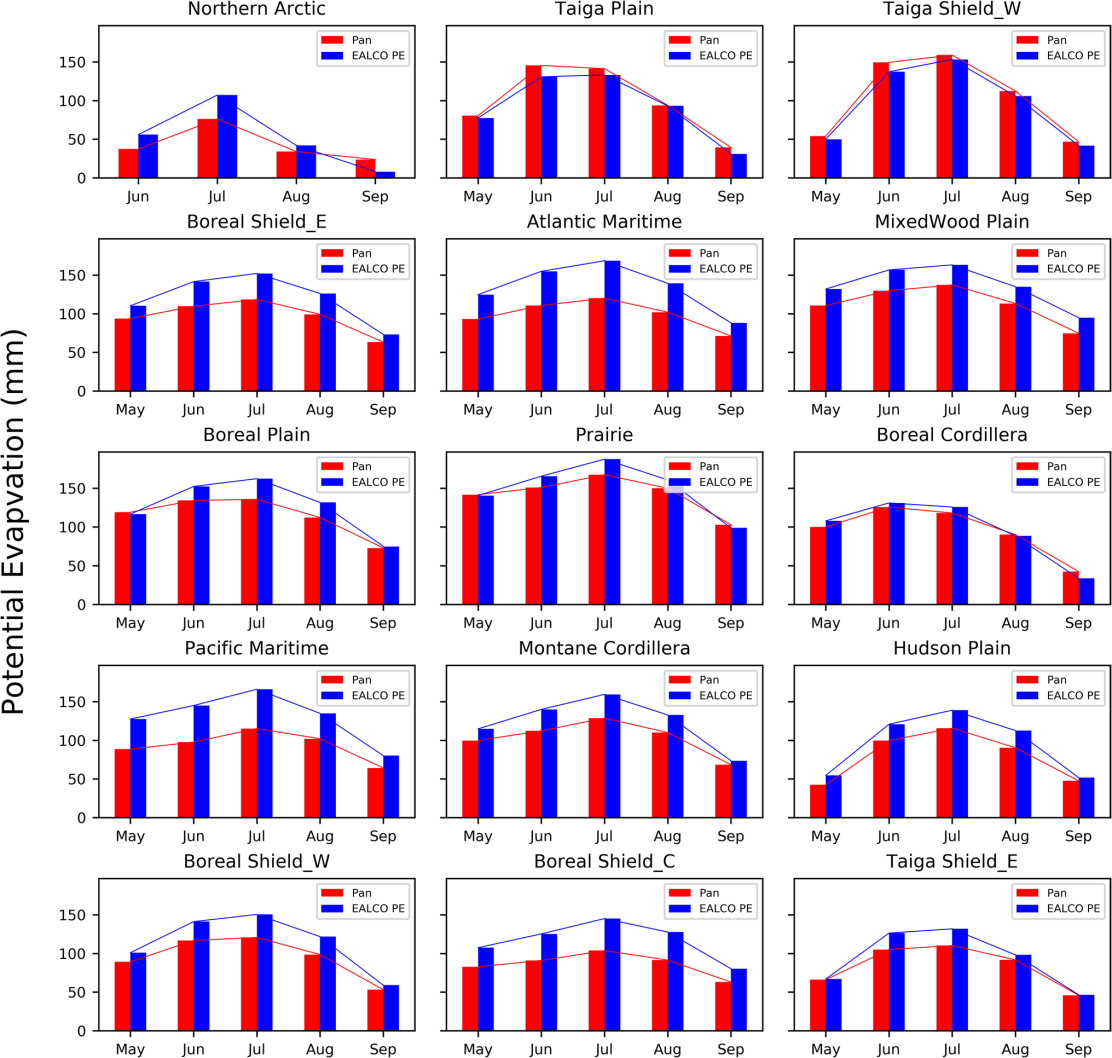
The seasonal variations of monthly EALCO PE (mm) and Pan evaporation (E_pan_, mm) in May (or June in Northern Arctic) to September 1979 -2007 in 15 ecozones of Canada.

The EALCO PE values at the pixels containing the E_pan_ sites for the days corresponding to the E_pan_ observations between May (or June in Northern Arctic) and September are extracted and averaged in the 15 ecozones for the comparisons of daily values (Fig. 2), seasonal variations (Fig. 3), and long-term trends (Table 1).

**Table 1:** The trends of EALCO PE and E_pan_ in the 15 ecozones in warm seasons, 1979–2007 (warm seasons refer to June–September in Northern Arctic and May–September in the other ecoregions, respectively, and * indicates the trend is significant at the 95% confidence interval).

Ecozone	Trend of E_pan_	Slope of E_pan_ (mm/season)	Trend of PE	Slope of PE (mm/season)	Periods
Northern Arctic	Increasing	0.17	Increasing	0.30	1979–2004
Taiga Plain	Increasing	1.85	Decreasing	− 0.61	1979–2003
Taiga Shield_W	Decreasing*	− 6.65	Decreasing	− 1.39	1979–1996
Taiga Shield_E	Increasing	3.40	Decreasing	− 1.06	1979–1995
Boreal Shield_E	Increasing	1.95	Increasing	2.01	1979–2005
Atlantic Maritime	Increasing*	2.65	Increasing*	− 3.46	1979–1999
MixedWood Plain	Decreasing	− 0.59	Decreasing*	− 1.85	1979–1998
Boreal Plain	Decreasing	− 0.96	Decreasing*	− 3.08	1979–2007
Prairie	Decreasing	− 0.42	Decreasing	− 2.07	1979–2007
Boreal Cordillera	Decreasing*	− 5.45	Decreasing	− 1.87	1979–1999
Pacific Maritime	Increasing*	5.10	Decreasing	− 0.49	1979–1999
Montane Cordillera	Decreasing*	− 3.84	Decreasing*	− 8.06	1979–2004
Hudson Plain	Decreasing*	− 4.15	Decreasing*	− 3.02	1979–2004
Boreal Shield_W	Decreasing	− 2.06	Decreasing*	− 7.31	1979–2007
Boreal Shield_C	Decreasing*	− 3.68	Decreasing*	− 0.36	1979–1999

The agreement between daily EALCO PE and E_pan_ varies among different climate (Fig. 2). The correlation coefficient (r) changes from 0.83 in Canadian Prairies to 0.47 in the Northern Arctic. The smallest difference between the two daily datasets is in Prairies indicated by an RMSE value of 0.98 mm, while the largest is in the Northern Arctic, shown as an RMSE value of 1.64 mm. The majority of EALCO PE data are larger than E_pan_, indicated by the modes of the histograms. EALCO PE data generally has one mode at 4 to 5 mm, and E_pan_ typically has one mode at 2–4 mm, except for the Taiga Plain and Taiga Shield West having multiple modes.

The EALCO PE and E_pan_ demonstrate the same seasonal variations with a peak in July (Fig. 3). The values of monthly EALCO PE data are comparable to E_pan_ in the Taiga Plain, Taiga Shield West, and Boreal Cordillera, but larger than E_pan_ in the other ecozones, especially in the Atlantic and Pacific Maritime, and Boreal Shield coast.

The EALCO PE and E_pan_ show the same trend directions (increasing/decreasing) in most of the ecozones but varying with changing rates (Table 1). The Taiga Plain, Taiga Shield_E, and Pacific Maritime are the only exceptions where the two datasets' slope is in the opposite direction. Nevertheless, neither the change of EALCO PE nor E_pan_ is statistically significant in the Taiga Plain and Taiga Shield_E. The Pacific Maritime thus is the only one where the trends of the two datasets are significantly different. The inconsistent trends are likely because meteorological conditions at a site level in Maritime climate may not be represented by the EALCO PE calculated based on gridded climate forcing fields at a 10 × 10 km^2^ grid.

### Spatial variations of PE

**Figure 4:**
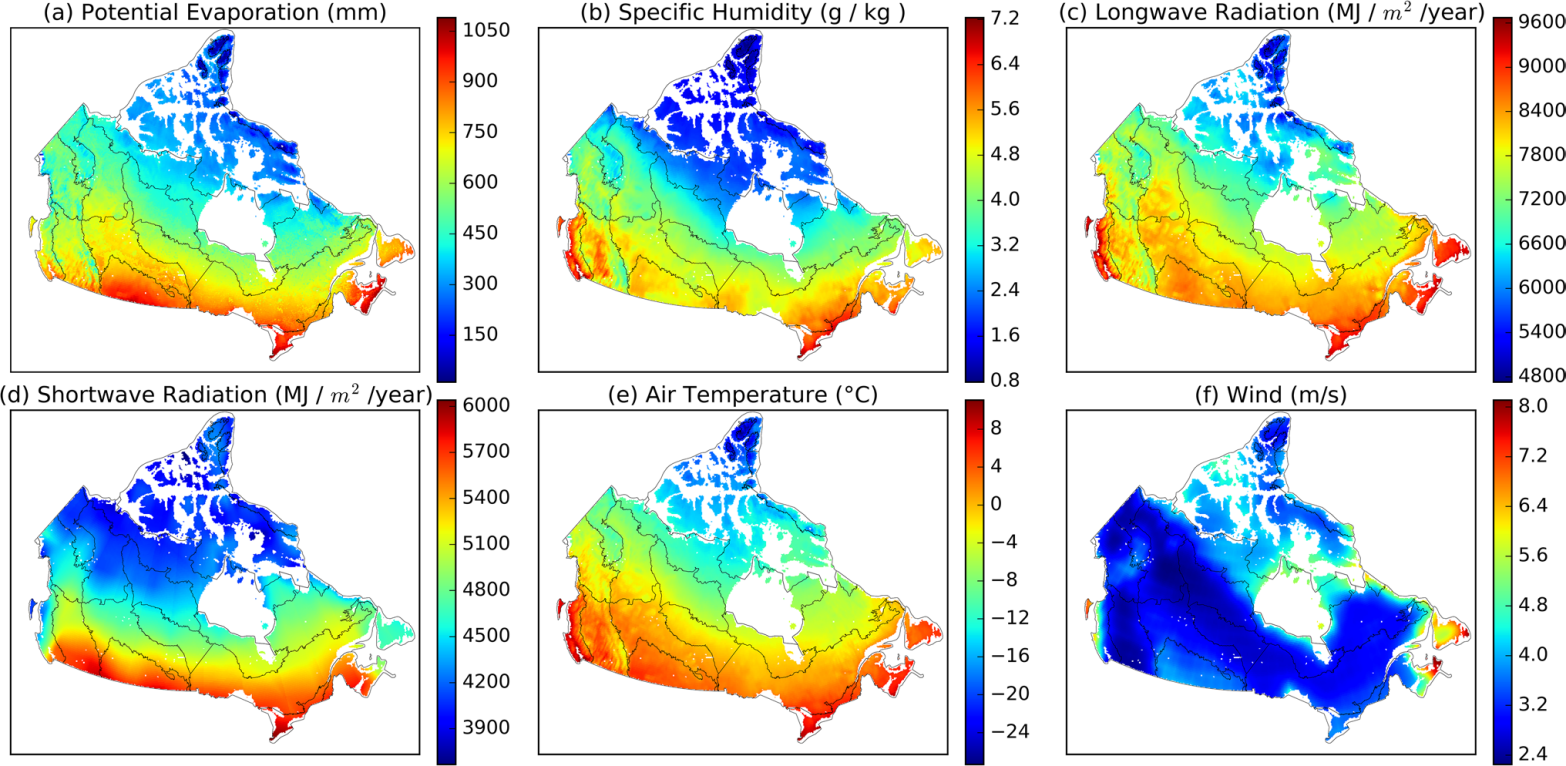
The spatial distribution of mean annual (**a**) potential evaporation (PE), (**b**) specific humidity (g/kg), (**c**) downward longwave radiation (MJ/m^2^/year), (**d**) downward shortwave radiation (MJ/m^2^/year), (**e**) air temperature (°C), and (**f**) wind speed (m/s) in 1979–2016 in Canada (The black lines depict the spatial extent of each ecozone. The map was created using our own script in Anaconda Python 3.8 downloaded from https://docs.anaconda.com/anaconda/install/).

Annual PE across Canada varies dramatically, decreasing from over 1000 mm in the MixedWood Plain to around 100 mm in the Arctic Cordillera (Fig. 4a). The 400 mm contour of annual PE largely coincides with the Canadian treeline. High annual PE (750–1050 mm) occurring in southern Canada and the East Coast responds to the high air temperature and downward shortwave and longwave radiation (Fig. 4c–e). High wind speed also accounts for the high PE in East Coast (Fig. 4f). Low annual PE (< 400 mm) occurs in the Arctic region as a response to low air temperature and downward shortwave and longwave radiation (Fig. 4c–e).

**Figure 5:**
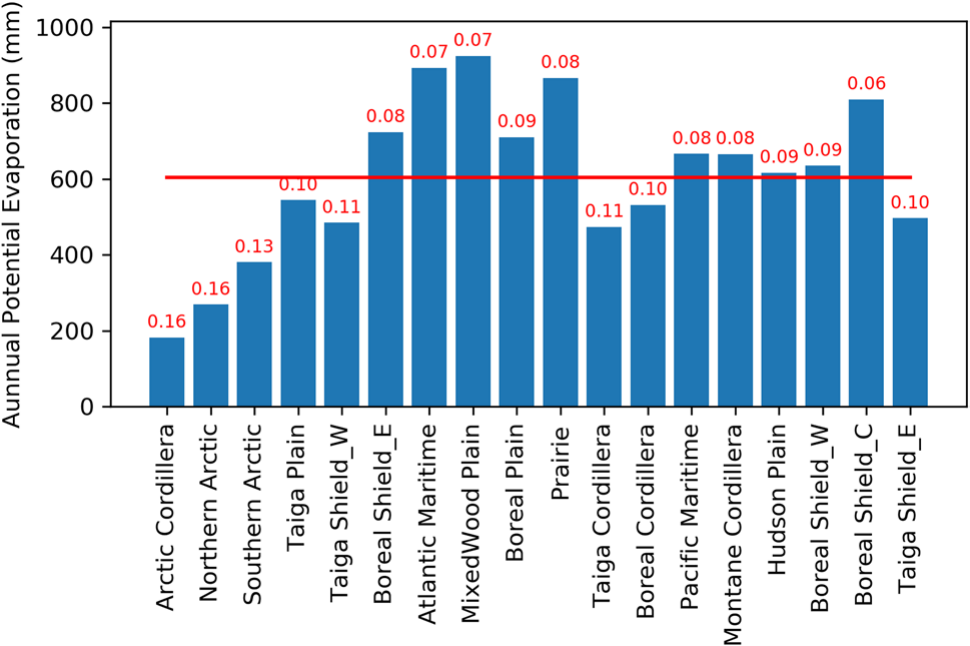
A 38-year (1979–2016) mean annual potential evaporation (PE) with the coefficient of variation (CV, red font) in the 18 ecozones across Canada (the red line marks the average PE in Canada).

The mean annual PE is aggregated for each ecozone (Fig. 5). The MixedWood Plain has the highest annual PE (924 mm), followed by the Atlantic Maritime, Prairie, and Boreal Shield coast, which all have annual PE over 800 mm. The Northern Arctic, Southern Arctic, and Arctic Cordillera have annual PE < 400 mm. The Arctic Cordillera has the lowest annual PE (181 mm) among all the ecozones. The average annual PE in Canada is around 600 mm.

The spatial variations of annual PE in each ecozone are indicated by the coefficient of variation (CV) in Fig. 5. The Arctic Cordillera and Northern Arctic have the most considerable spatial variation in PE among the 18 ecozones, followed by the Northern and Southern Arctic. PE in Boreal Shield Coast has the least spatial variation.

### Seasonal variations of PE in Canada

**Figure 6:**
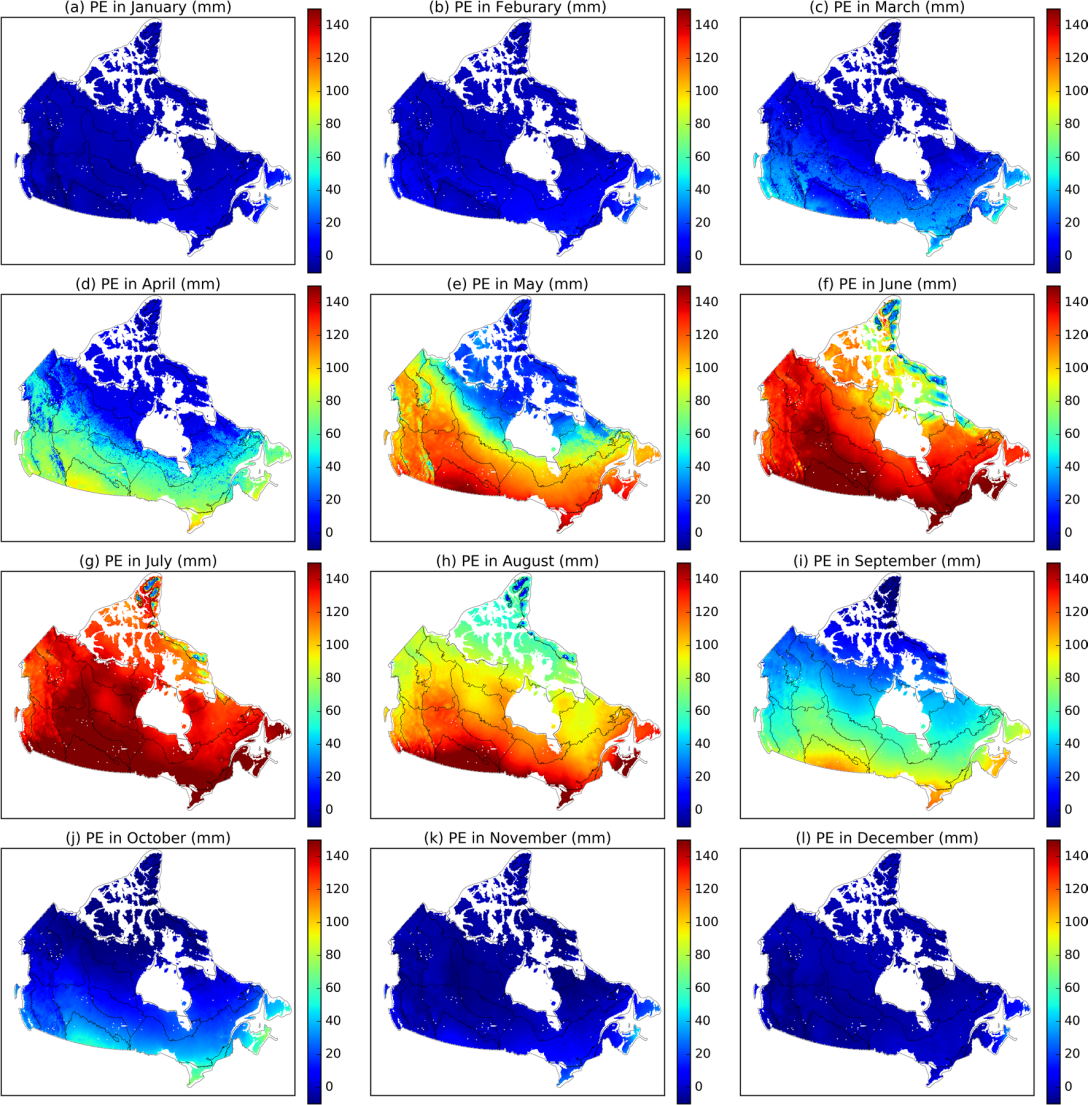
The spatial and seasonal variations in mean monthly potential evaporation (PE, mm) during 1979–2016 in Canada (The black lines depict each ecozone's spatial extent. The map was created using our own script in Anaconda Python 3.8 downloaded from https://docs.anaconda.com/anaconda/install/).

In warm seasons, PE generally increases from May to July and then decrease from July to September (Fig. 6), in response to the seasonal change in air temperature and shortwave solar radiation. In cold seasons, the mean monthly PE is low (< 100 mm). Negative monthly PE occurs in the Arctic regions, indicating that EALCO simulated condensation exceeds PE. However, monthly PE in the East Coast and the MixedWood Plain remains positive due to relatively high air temperature and shortwave radiation.

The long-term average and CV of monthly PE indicate dramatic seasonal changes in each ecozone (Table 2). From January to July, PE across Canada gradually increases and reaches a peak in July (or June in Taiga Cordillera and Boreal Cordillera). The peak values vary with regions, with the largest (183 mm) in the Prairie and the smallest (90 mm) in the Arctic Cordillera. Indicated by CV, the seasonal variation in PE decreases from the Arctic Cordillera, Northern Arctic, and Southern Arctic to the Taiga Plain, Taiga Shield West, Taiga Shield West, Taiga Shield East, Taiga Cordillera, and Boreal Cordillera. The smallest seasonal variation of PE occurs in the Pacific Maritime, Atlantic Maritime, and MixedWood Plain. The Oceans and Great Lakes retain energy in warm seasons and release it in cold seasons, accounting for the small seasonal variation in PE.

**Table 2:** The seasonal changes in potential evaporation (PE) over the 18 Ecozones, shown by monthly PE and the coefficient of variation (CV) (Bold font highlights the maximum monthly PE).

Ecozones	Monthly PE (mm)												CV
	Jan	Feb	Mar	Apr	May	Jun	Jul	Aug	Sept	Oct	Nov	Dec	
Arctic Cordillera	− 1.8	− 0.7	0.3	3.8	16.4	51.7	**90.1**	53.0	4.9	− *5.5*	− 4.9	− 2.9	1.8
Northern Arctic	− 1.6	− 0.8	0.5	4.4	20.2	91.7	**122.6**	70.1	12.5	*− 7.7*	− 5.4	− 3.0	1.7
Southern Arctic	− 2.1	− 1.1	0.9	6.8	33.9	112.5	**137.9**	93.2	31.5	− 5.1	*− 6.7*	− 3.9	1.6
Taiga Plain	− 2.0	− 0.1	7.8	33.2	97.2	139.2	**142.0**	101.9	43.2	− 0.7	*− 5.2*	− 3.4	1.3
Taiga Cordillera	− 2.7	− 1.3	1.4	18.1	81.9	***131.3***	129.2	87.5	28.9	*− 6.1*	− 4.6	− 3.6	1.4
Taiga Shield_W	− 2.5	− 0.8	5.0	20.2	72.0	128.5	**142.9**	102.3	42.7	− 1.7	*− 8.3*	− 4.3	1.4
Taiga Shield_E	− 1.0	1.7	9.0	25.6	68.3	119.6	**130.2**	96.5	47.1	10.5	− 5.6	− 4.2	1.2
Boreal Cordillera	− 3.1	− 0.1	9.1	42.7	102.4	***129.2***	126.1	92.0	39.1	0.4	− 5.2	− 5.7	1.2
Boreal Plain	− 3.0	1.3	18.7	64.9	122.3	149.8	**158.9**	130.4	73.7	22.0	− 5.1	− 5.9	1.1
Hudson Plain	− 0.4	4.0	16.1	41.3	93.8	129.7	**139.2**	114.1	62.5	19.8	− 1.2	− 3.5	1.1
Boreal Shield_W	− 2.9	2.1	16.5	50.6	108.0	142.0	**150.8**	121.4	63.2	13.4	− 6.5	− 6.1	1.1
Montane Cordillera	− 5.3	3.0	29.2	65.4	110.6	134.7	**146.3**	118.4	63.1	19.0	− 4.6	− 7.9	1.0
Prairie	− 3.8	− 0.6	17.7	80.4	138.5	163.1	**183.3**	156.0	96.5	41.2	1.7	− 5.7	1.0
Boreal Shield_E	2.4	8.6	27.6	61.3	111.4	139.9	**147.1**	120.9	72.9	28.9	3.5	− 1.1	0.9
Pacific Maritime	− 0.8	8.2	34.9	67.5	107.3	125.2	**135.5**	108.4	60.3	22.6	0.2	− 4.4	0.9
Atlantic Maritime	6.2	12.7	32.7	68.0	124.6	153.8	**168.2**	142.1	92.1	48.2	16.0	4.9	0.8
MixedWood Plain	1.0	7.9	34.2	85.4	134.7	159.1	**167.8**	141.9	102.1	56.1	17.9	− 0.1	0.8
Boreal Shield_C	13.0	18.0	35.9	66.9	104.1	128.2	**146.3**	126.8	81.7	42.1	20.1	11.3	0.8

### Long-term trends of PE in Canada

**Figure 7:**
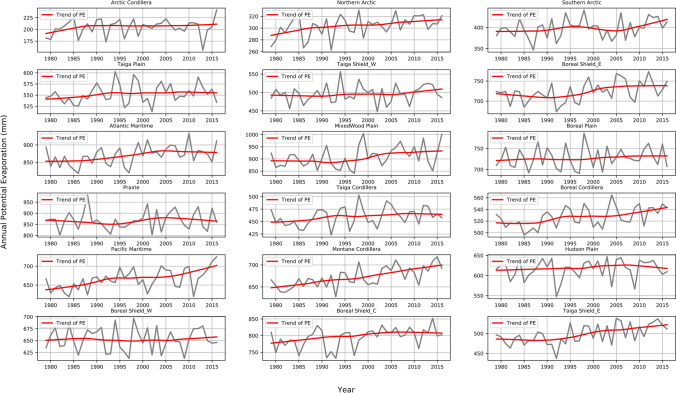
The temporal profile of annual potential evaporation (PE, mm) and the LOESS regression trends in 1979–2016 in the 18 ecozones in Canada.

Annual PE generally varies in a monotonic manner from 1979 to 2016, with an increasing or no trend across Canada (Fig. 7). An increasing PE trend is observed in the Northern Arctic, Atlantic Maritime, MixedWood Plain, Taiga Cordillera, Boreal Cordillera, Montane Cordillera, Pacific Maritime, Boreal Shield Coast, and Taiga Shield East. Some regions, such as the Boreal Cordillera, Taiga Shield East, and the Boreal Shield East, exhibit a turning point of no trend to an increasing trend around 1990. The increasing trends of these ecozones correspond to the elevated air temperature (Supplementary Fig. S1). However, an increase in air temperature does not necessarily lead to an increase in PE in some ecozones, such as Taiga Shield West (Supplementary Fig. S1).

**Figure 8:**
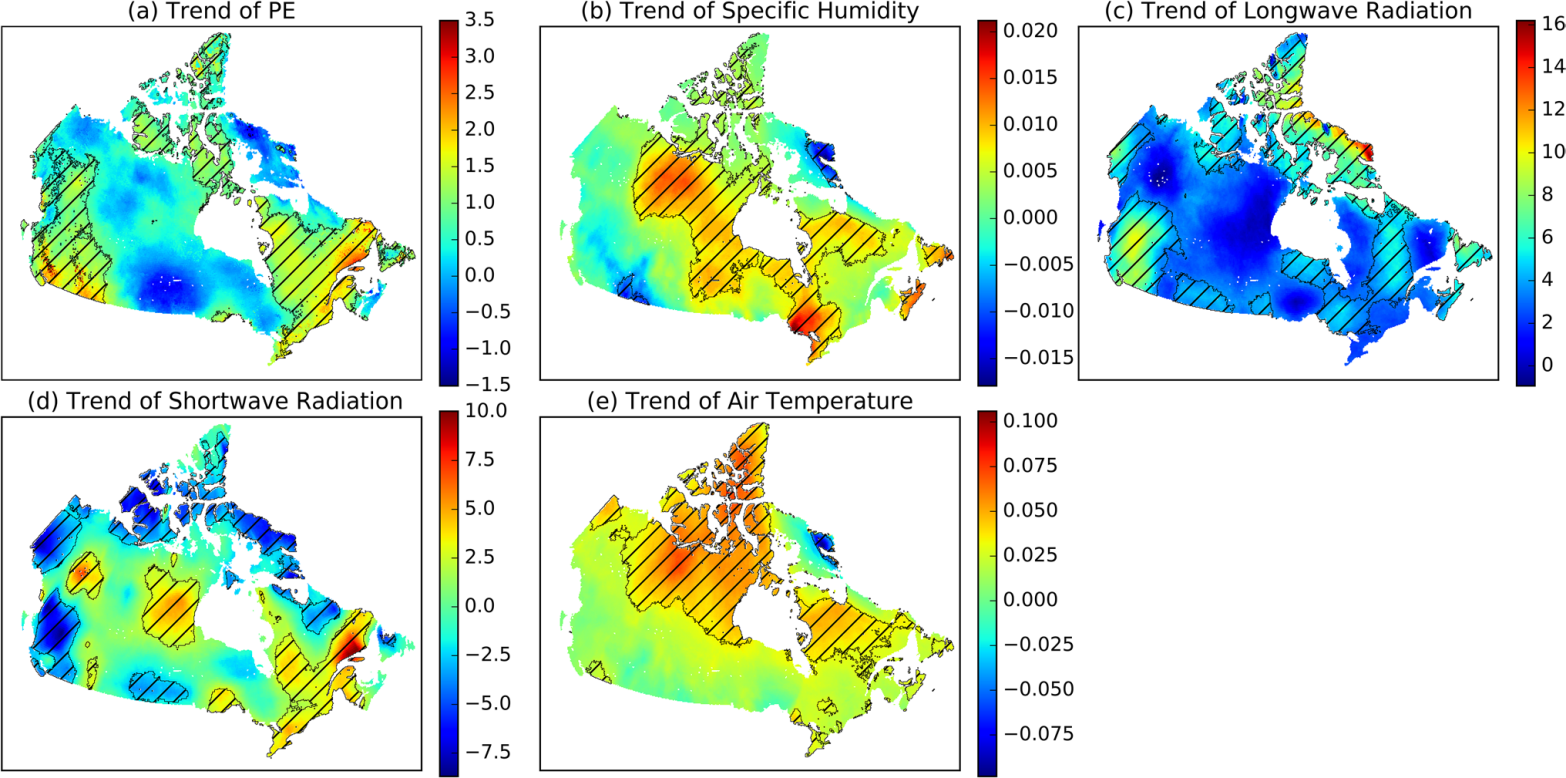
The trends of annual potential evaporation (PE) and climate variables in 1979–2016 in Canada indicated by the Sen's slope of (**a**) PE (mm/year), (**b**) specific humidity (g /kg/year), (**c**) downward longwave radiation (MJ/m^2^/year), (**d**) downward shortwave radiation (MJ/m^2^/year), and (**e**) air temperature (°C/year) (Hatching indicates that the trends are significant at the 95% confidence interval. The map was created using our own script in Anaconda Python 3.8 downloaded from https://docs.anaconda.com/anaconda/install/).

Increasing annual PE trends are observed in the Northern Arctic, East Canada, and West Canada at a rate smaller than 4 mm/year (Fig. 8). The increasing trends are related to different climate scenarios. In the Northern Arctic, the increase of annual PE is mainly associated with the elevated air temperature (Fig. 8e) and downward longwave radiation (Fig. 8c). The increase of annual PE in West Canada is primarily related to the increased downward longwave radiation (Fig. 8c, d) and wind speed in the Pacific Maritime. The increase of annual PE in East Canada is mainly attributed to the increase in downward shortwave and longwave radiation (Fig. 8d). Elevated air temperature also plays a role in the increase of PE in some regions of East Canada, such as the Taiga Shield East (Fig. 8e). The wind speed trend is not shown in Fig. 8 because the changes are only observable in a few regions, and the change rate is meager (< 0.01 m/s/year).

The annual PE trends are not statistically significant at the 95% confidence interval in the other regions of Canada, including Taiga Plain, Taiga Shield West, Hudson Plain, Boreal Shield West. The non-significant trends are related to the increased humidity (Fig. 8b), which weakens the aerodynamic component and decreases PE. The decreased PE offsets the increase in PE associated with the increased shortwave radiation and air temperature (Fig. 8d, e).

**Figure 9:**
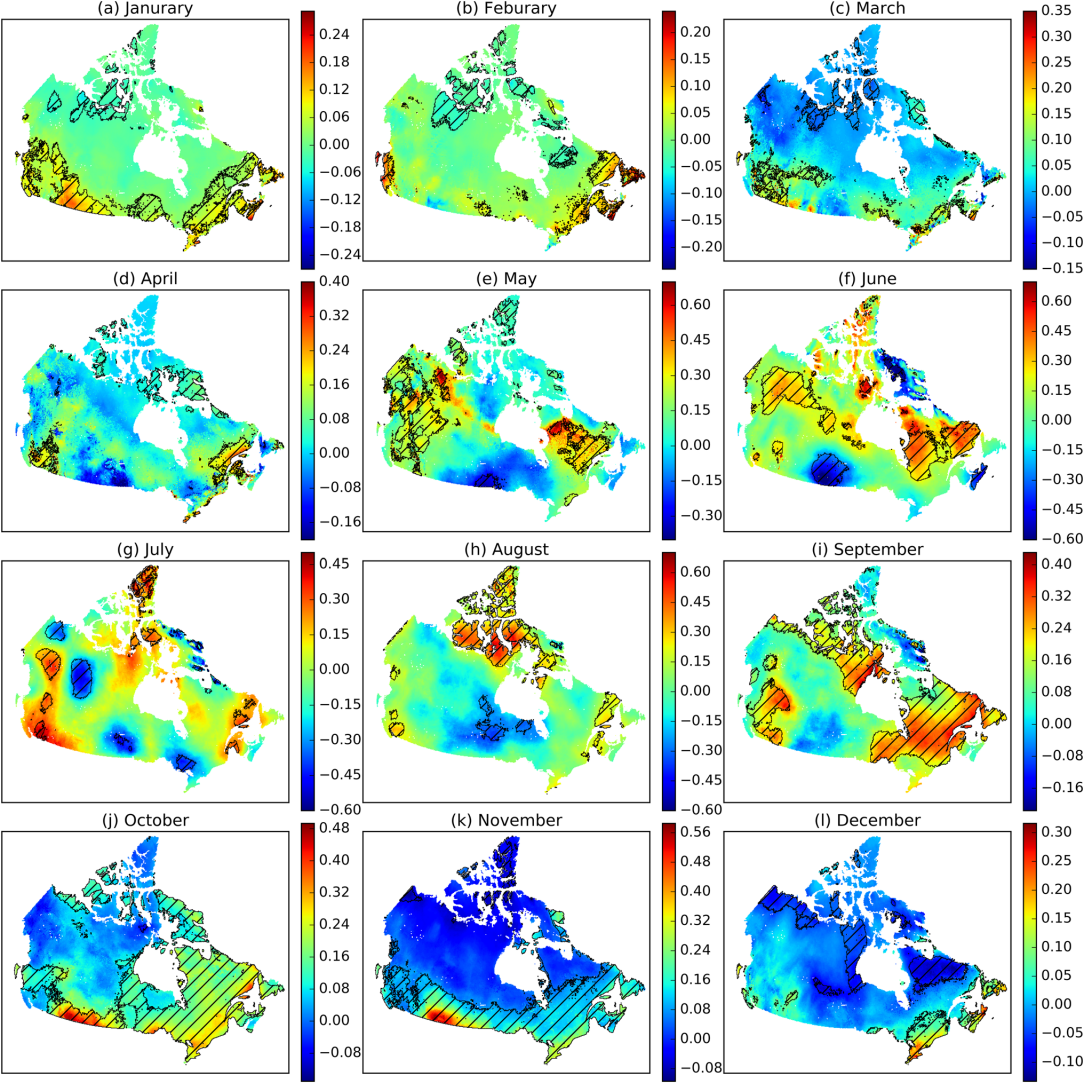
The trends of monthly potential evaporation (PE, mm/month) in 1979–2016 in Canada, indicated by Sen's slope (Hatching indicates the statistically significant trend at the 95% confidence interval. The map was created using our own script in Anaconda Python 3.8 downloaded from https://docs.anaconda.com/anaconda/install/).

Increasing trends of monthly PE are observed in both warm and cold seasons (Fig. 9). In warm seasons, the Northern Arctic, East Canada, and West Canada (Fig. 9e–i) have experienced an increase in PE. Specifically, the Northern Arctic has increasing PE in July to September, corresponding to the elevated air temperature and downward longwave radiation (Supplementary Figs. S2, S4). PE in East Canada has an increasing trend in May, June, July, and September, mainly due to the increase of air temperature (Supplementary Fig. S2). West Canada has an increasing PE in May, July, and September, corresponding to the increased downward shortwave solar radiation in May (Supplementary Fig. S3), downward longwave radiation in July (Supplementary Fig. S4), and augmented wind speed in September (Supplementary Fig. S5), respectively. In cold seasons, East Canada has an increasing PE in November to February, associated with the elevated downward longwave radiation (Supplementary Figs. S3, S4). Southern Canada has an increasing PE in January, October, and November, as a response to the increase in downward longwave radiation and wind speed (Supplementary Fig. S5).

Decreasing trends of monthly PE are found mainly in warm seasons. A decrease in PE is observed in the Prairie in May to July, Taiga Cordillera, Taiga Plain, and Boreal Shield East in July, and Hudson Plain and Boreal Shield West in August (Fig. 9). Such a decrease is mainly associated with an increase in specific humidity (Supplementary Fig. S6). In Prairie, a significant decrease in PE occurs from May to July. The decrease of PE in warm seasons is offset by the increase of PE in cold seasons, accounting for the non-significant decreasing trend of annual PE in Prairie (Fig. 8).

## Discussion

Analyzing the spatial variations of annual PE in cold regions has focused on warm seasons because no data is available in cold seasons^30,31^. Our study generated a daily dataset for both warm and cold seasons in Canada and investigated the spatial and seasonal variations and temporal trends of annual and monthly PE. The calculated total PE in cold season accounts for 7–26% of annual PE across Canada, indicating PE in cold seasons may not be negligible in investigating the terrestrial water cycle's response to climate change.

EALCO PE is generally higher than E_pan._ The finding is consistent with the conclusion in Northern America^31^ and Spain^24^. The difference between the two datasets likely results from many factors. A potential source of the difference could be the errors of the E_pan_ data, including some extremely high values (Fig. 2), which are caused by multiple factors, such as intensive rain, high wind speeds^32^, and errors introduced while taking readings^33,34^. Also, *K*_pan_ (0.77) used for E_pan_ correction in this study is likely too small for some ecozones. A *K*_pan_ of 0.78 to 0.94 was suggested in the humid Atlantic Maritime by Xing et al. (2008)^35^. The smaller *K*_pan_ may account for the more considerable discrepancy in EALCO PE and E_pan_ in the Atlantic Maritime, Boreal Shield Coast, Pacific Maritime, and MixedWood Plain than the other ecozones. *K*_pan_ varies from an arid to a humid environment^36^, and therefore applying one *K*_pan_ to all the ecozones across Canada is not ideal. In the future, *K*_pan_ should be further evaluated in different climates, because it is the most significant contributor to the uncertainties of E_pan_^37^. Besides, albedo used for EALCO PE calculation is smaller than Pan albedo, accounting for the larger values of EALCO PE than E_pan_. The water albedo used in EALCO is 0.05, while the Pan albedo is around 0.14^38–41^. It is also worth noting that EALCO PE calculation did not incorporate the ground heat flux (G) into the energy component, which might affect daily PE estimation^42,43^. However, the effects of G on PE estimation significantly decrease at a monthly scale and are neglected at an annual scale^43,44^. Hence, the omission of G has little influence on the spatial variations and trends of annual PE in this study.

The difference in EALCO PE and E_pan_ changes among climates. It might indicate that the Penman equation's applicability for PE estimation varies in different environments; however, it is difficult to conclude. The potential errors in E_pan_, the fixed value of *K*_pan_, and the albedo difference contribute to the discrepancy between the two datasets, as discussed above. The fewer pan evaporation sites may also account for the smaller r and larger RMSE values in the Northern regions. In this case, we found that it is difficult to understand the varying discrepancy mechanism in a diverse environment without further monitoring and modeling.

The spatial variations of annual PE are consistent with the distribution of downward shortwave radiation and air temperature, indicating the significant role of energy in determining PE in Canada. This finding is different from Spain in the Mediterranean climate, where the spatial variations of PE match the distribution of mean air temperature, relative humidity, and cloudiness^24^. The aerodynamic component, including wind and specific humidity, is also essential for determining PE, provided sufficient energy is available. For instance, the Boreal Shield Coast has lower downward shortwave radiation and air temperature but higher annual PE than the Pacific Maritime (Fig. 4). The larger PE in the Boreal Shield Coast is associated with the higher wind speed and lower specific humidity that accelerate evaporation through enhancing the transfer of water vapor from a moist surface (or air) to dry air.

This study shows that increasing PE responds to the increase in the energy component, including downward shortwave and longwave radiation, and the aerodynamic component, specifically, the air temperature. The air temperature was deemed to play a less important role than solar radiation, vapor pressure deficit, and wind speed on accounting for the trends of E_pan_^4,38,39,45^. However, this study shows that the elevated air temperature plays a vital role in the change of annual PE in the Northern Arctic and East Canada (Fig. 8) and the increase of monthly PE in winter in Southern Canada (Fig. 9). The vital role of air temperature was also discussed by Azorin-Molina (2015)^24^ and Wang et al. (2018)^15^. Wind speed was hypothesized to have an essential role in evaporation change globally^9,45^. This study shows that wind speed plays a limited role in annual PE changes in vast Canada.

## Conclusion

This study fills the PE data gap in cold season over Canada's landmass by generating a daily PE dataset using the EALCO that integrates the evolutions of water–ice–snow into the estimation. Despite the difference in values, the EALCO PE and E_pan_ exhibit similar seasonal variations and trends in most areas.

The modeled annual PE in Canada is around 600 mm/year, decreasing from about 1000 mm/year in southern Canadian prairies, southern Ontario, and East Coast to less than 100 mm/year in the northern Arctic. The spatial variations in annual PE generally match the spatial distributions of air temperature and downward shortwave solar radiation. The maximum monthly PE is in July in vast Canada, with the largest seasonal variations in the Northern Arctic and the smallest in the East Coast, West Coast, and southern Ontario.

Annual PE shows an increasing trend in the regions of the Northern Arctic, West, and East Canada at a rate of 1.5–4 mm/year. The increase in the Northern Arctic is a response to the elevated air temperature and downward longwave radiation. In West Canada, the increase in annual PE corresponds to the augmented downward longwave radiation and wind speed. The increase in East Canada is mainly related to the increase in downward shortwave and longwave radiation and air temperature. The increasing annual PE trends is primarily accounted for by the ascending PE in warm seasons.

## Methods

### Potential evaporation data

The PE dataset was generated using the Ecological Assimilation of Land and Climate Observation (EALCO) model. EALCO is developed for simulating physical, physiological, and biogeochemical processes in terrestrial ecosystems using in situ and remote sensing observations. The model has five major modules for simulating land surface radiation transfer, energy balance, water dynamics, and carbon and biogeochemical nitrogen cycles^28,46,47^. The robustness of the EALCO model has been tested in diverse ecosystems in many modeling studies, including the Boreal Ecosystem-Atmosphere Study (BOREAS)^48,49^, the AmeriFlux Network^50^, the Fluxnet Canada Research Network (FCRN)^51–53^, and the Free-Air CO_2_ Enrichment (FACE) Model-Data Synthesis study^54–57^. Recently, actual evapotranspiration simulated using EALCO was compared with Fluxnet in situ observations, and MODIS evapotranspiration products, and the simulations of other land surface models, including the Variable Infiltration Capacity (VIC) and Community Land Model (CLM), for entire Canada's landmass^58^. The actual evapotranspiration was also assessed by examining water budget closures for all gauged watersheds in Canada^59–61^. Developed in Canada, the model has comprehensive algorithms, particularly for simulating energy and water transfer processes in cold regions.

The PE algorithm in EALCO integrates the dynamic surface evolutions of water, ice, and snow into the Penman equation ^1^.

1 $$PE = \frac{{\Delta R_{n} }}{{\left( {\Delta + \gamma } \right)\lambda }} + \frac{\gamma }{{\left( {\Delta + \gamma } \right)}}\frac{{6.43f\left( u \right)\left( {e_{s} - e_{a} } \right)}}{\lambda }$$

where PE is the daily potential evaporation (mm day^−1^), $\Delta$ is the slope of the saturation vapor pressure curve (kPa °C^−1^), R_n_ is net radiation (MJ m^−2^ day^−1^), $f\left( u \right)$ is a wind function,$e_{s}$ and $e_{a}$ are saturated and actual vapor pressure (kPa), respectively, γ is psychrometric constant (kPa °C^−1^), calculated using Eq. (2), and $\lambda$ is the latent heat of water vaporization (for a liquid water surface) and fusion (for a snow or ice surface) (MJ kg^−1^).

2 $$\gamma = \frac{{C_{p} P}}{\varepsilon \lambda }$$

where $C_{p}$ is the specific heat at constant pressure (MJ kg^−1^ °C^−1^), P is atmospheric pressure (kPa), and $\varepsilon$ = 0.622, is the ratio of molecular weight of water vapor to dry air.

The first term in Eq. (1) represents the radiative energy control on liquid water evaporation or snow and ice sublimation. The net radiation R_n_ is calculated as:

3 $$R_{n} = \left( {1.0 - \alpha } \right)R_{dsw} + R_{dLw} - R_{uLw}$$

where $R_{dsw}$ is downward shortwave radiation, $R_{dLw}$ and $R_{uLw}$ are downward and upward longwave radiation, respectively, and $\alpha$ is the surface albedo. In warm seasons with a water surface, the value for *α* is 0.05. In cold seasons with ice and snow surfaces, the *α* in EALCO is modeled using multiwavelength and multi-direction radiation algorithms^28^. The modeled *α* values reflect the dynamic changes of *α* as the variations in weather conditions, solar zenith angle, and the snow/ice processes of aging, melting, refreezing, metamorphism, and densification^28,29^. The onset time for surface change between frozen and thaw are referenced to the corresponding simulations in EALCO for the top 10 cm soil layers.

The second term in Eq. (1) represents the aerodynamic control of PE. In this component, the wind function $f\left( u \right)$ for an open water surface was given by $f\left( u \right) = 0.54{\text{u}} + 0.5$^1^, and u is the wind speed at 2 m height. The $e_{s}$ and ∆ are calculated using Eqs. (4) and (5).

4 $$e_{s} = \left\{ {\begin{array}{*{20}l} 0.1*6.10588*e^{{\left( {17.32491*\frac{{T_{a} }}{{(T_{a} + 238.102)}}} \right)}} & T_{a} \ge 0^\circ C \\ 0.1*6.10588*e^{{\left( {21.874*\frac{{T_{a} }}{{T_{a} + 265.5}}} \right)}} & T_{a} < 0^\circ C \\ \end{array} } \right.$$

5 $$\Delta = \left\{ {\begin{array}{*{20}l} 238.102*17.32491*\frac{{e_{s} }}{{\left( {T_{a} + 238.102} \right)^{2} }} & T_{a} \ge 0^\circ C \\ 265.5*21.874*\frac{{e_{s} }}{{\left( {T_{a} + 265.5} \right)^{2} }} & T_{a} < 0^\circ C \\ \end{array} } \right.$$

where $T_{a}$ is the air temperature at 2 m height (°C).

PE was calculated at a 10 km resolution and a daily time step over Canada's landmass using the global meteorological forcing data produced by Sheffield et al. (2006)^62^ and downloaded from https://hydrology.princeton.edu/data.pgf.php. The meteorological forcing data used in the EALCO simulation includes downward shortwave and longwave radiation, wind speed, air temperature, precipitation, specific humidity, and atmospheric pressure. The datasets are available at 0.25-degree spatial resolution and were downscaled to 10 × 10 km^2^ grids using bilinear interpolation for this study^47^. Precipitation was treated as rain when $T_{a}$ is equal to or above 0 °C and snow while $T_{a}$ is below 0 °C in the model.

### Pan evaporation data

E_pan_ data were measured at 260 weather stations across Canada in 1960–2007 by Environment and Climate Change Canada (ECCC). The data record at each station varies from one to 46 years, and most measurements were available from May to September. In this study, E_pan_ measured at 141 sites in 1979–2007 overlapping with the period of the EALCO PE were used for comparisons (Fig. 1; Supplementary Table S1). The EALCO PE values at the pixels containing the E_pan_ sites for the days corresponding to the E_pan_ observations in May–September are extracted and averaged in the 15 ecozones to compare daily values, seasonal variations, and trends.

The Pan evaporation data were measured using Class-A Pan evaporimeter, which has a diameter of 120 cm and a depth of 25 cm. The Pan evaporimeter is mounted on an open wooden platform to allow air circulation. E_pan_ measurements are affected by the sensible heat transfer between air and water through the sides and bottom of a pan^33,34^. A pan coefficient (*K*_pan_) is typically used to correct E_pan_ to minimize the heat transfer effects. In previous studies, *K*_pan_ = 0.70, 0.77, and 0.80 were used for estimating open-water evaporation in Canada^30^, PE estimation in conterminous North America^31,63^, and irrigation estimation in moderate humidity and wind condition^64^ , respectively. In this study, *K*_pan_ = 0.77 was used to be consistent with the coefficient used by Linacre (1994)^63^ and Hember et al. (2017)^31^. After the correction, the E_pan_ data having a value out of three standard deviations from the mean were removed using the Python script before the data were compared with EALCO PE.

### Trend analysis

The long-term trends of annual and monthly PE in 1979–2016 were analyzed using the non-parametric Mann–Kendell (M–K) test^65,66^. Sen's slope indicates the trends, and the statistically significant trends were identified at the 95% confidence interval (*p* < 0.05). The temporal trends of climate variables, including annual air temperature, downward shortwave and longwave radiation, wind speed, and specific humidity, were also analyzed to investigate the climate drivers for the PE changes. Since the M–K test and the Sen's slope are only applicable to time series datasets with a monotonic trend, the temporal structure of the data was graphically examined using the non-parametric locally weighted regression (LOESS) to check the validity of the M–K test for the PE dataset^67^.

## Supplementary Information

Supplementary Information.
