# Supplementary material for: Spatial variations and long-term trends of potential evaporation in Canada
Source: Sci Rep. 2020 Dec 16;10:22089. doi: 10.1038/s41598-020-78994-9 (PMC7744546; doi:10.1038/s41598-020-78994-9)
Supplement: Supplementary file 1 — Supplementary Information. [file 41598_2020_78994_MOESM1_ESM.pdf]

Supplementary Materials for

Spatial variations and long-term trends of potential evaporation in Canada

Zhaoqin Li[1], Shusen Wang[1*], and Junhua Li[1]

[1] Canada Centre for Remote Sensing, Natural Resources Canada, 560 Rochester Street, Ottawa, ON K1A 0E4, Canada

[*] Correspondence to shusen.wang@canada.ca

Introduction

The Supplementary Materials contain a list of the 141 Pan evaporation sites and their distribution in the ecozones used in this study (Table S1). The temporal profile of annual air temperature (°C) and the trends identified by the LOESS regression in 1979–2016 in the 18 ecozones are shown in Figure S1. The trends of monthly air temperature, downward shortwave radiation, downward longwave radiation, wind, and specific humidity over Canada's landmass are presented in Figures S2-S6, respectively.

Table S1: The List of the 141 Pan evaporation sites used in this study, with latitude (Lat) and longitude (Lon), start_year (Start_Y) and (End_Y), and start_month (Start_M) and end_month (End_M) of the Pan evaporation measurements, as well as the ecozone where each site locates. (Boreal Shield_C, Boreal Shield_E, and Boreal Shield_W refers to Boreal Shield Coast, Boreal Shield East, and Boreal Shield West, respectively)

Station_ID	Lat	Lon	Elev (m)	Start_Y	End_Y	Start_M	End_M	Ecozones
7021918	45.42	-71.63	212.8	1979	1980	5	9	Atlantic Maritime
7024280	45.37	-71.82	181	1979	1995	5	9	Atlantic Maritime
7027372	45.27	-71.52	393.5	1979	1981	5	9	Atlantic Maritime
7027516	45.75	-70.68	335	1979	1994	5	9	Atlantic Maritime
7027802	45.37	-71.53	345.9	1979	1985	5	9	Atlantic Maritime
7028676	46.38	-70.93	152.4	1979	1985	5	9	Atlantic Maritime
7028906	45.4	-71.3	512	1979	1985	5	9	Atlantic Maritime
7053649	48.28	-67.57	235.9	1979	1994	5	9	Atlantic Maritime
7055380	48.95	-65.52	574.5	1979	1993	5	9	Atlantic Maritime
7055420	48.03	-65.27	45	1979	1994	5	9	Atlantic Maritime
7055793	48.63	-68.13	15.2	1979	1984	5	9	Atlantic Maritime
7056480	48.45	-68.52	35.7	1979	1993	5	9	Atlantic Maritime
7056970	48.33	-68.05	323.1	1979	1985	5	9	Atlantic Maritime
8101000	47.01	-65.47	32.9	1979	1991	5	9	Atlantic Maritime
8101600	45.92	-66.62	39.6	1979	1999	5	9	Atlantic Maritime
8103256	47.42	-66.93	265.1	1979	1999	5	9	Atlantic Maritime
8202800	45.07	-64.48	48.8	1979	1996	5	9	Atlantic Maritime
8203700	45.77	-64.25	19.8	1979	1998	5	9	Atlantic Maritime
8205990	45.37	-63.27	39.9	1979	1997	5	9	Atlantic Maritime
8300400	46.25	-63.13	22.6	1979	1992	5	9	Atlantic Maritime
2100630	60.77	-137.58	595.3	1979	1983	5	9	Boreal Cordillera
2100700	63.62	-135.87	503.8	1979	1987	6	9	Boreal Cordillera
2100880	62.83	-137.31	445	1979	1999	5	9	Boreal Cordillera
2101300	60.71	-135.07	706.2	1979	1996	5	9	Boreal Cordillera
2202208	60.03	-111.93	204.1	1979	2003	5	9	Boreal Plain
3013247	53.45	-114.47	746.8	1979	1987	5	9	Boreal Plain

3064531	57.08	-111.58	310.3	1979	1981	5	9	Boreal Plain
3066001	55.3	-114.78	580.6	1979	1993	5	9	Boreal Plain
3070560	55.2	-119.4	744.9	1979	1996	5	9	Boreal Plain
4055085	52.82	-104.6	480.1	1979	1995	5	9	Boreal Plain
4075518	53.33	-104	371.9	1979	2004	5	9	Boreal Plain
4083321	52.82	-102.32	358.1	1979	1993	5	9	Boreal Plain
5031038	50.63	-97.02	222.8	1979	1991	5	9	Boreal Plain
5042425	50.7	-99.68	756.2	1979	1983	5	9	Boreal Plain
5052060	53.72	-101.53	262.1	1979	1985	5	9	Boreal Plain
8401700	48.95	-54.58	151.2	1979	1999	6	9	Boreal Shield_C
8403506	47.62	-52.74	140.5	1979	1979	5	9	Boreal Shield_C
8403800	48.53	-58.55	24.7	1979	1990	5	9	Boreal Shield_C
5030282	51.03	-95.7	259	1979	1984	6	9	Boreal Shield_E
5031320	49.62	-95.2	326.7	1979	2005	5	9	Boreal Shield_E
6014350	52.23	-87.88	254.5	1979	1988	5	9	Boreal Shield_E
6016525	51.45	-90.22	390.8	1979	1989	5	9	Boreal Shield_E
6020379	48.75	-91.62	395.3	1979	1988	5	9	Boreal Shield_E
6036904	49.65	-93.72	358.1	1979	1999	5	9	Boreal Shield_E
7012240	46.87	-71.65	166.1	1979	1985	5	9	Boreal Shield_E
7018574	46.9	-71.5	167.6	1979	1980	5	9	Boreal Shield_E
7035160	46.57	-75.55	244	1979	1994	5	9	Boreal Shield_E
7040440	49.13	-68.2	21.6	1979	1995	5	9	Boreal Shield_E
7042388	47.32	-71.15	640	1979	1995	5	9	Boreal Shield_E
7060825	50.73	-71.05	506	1979	1994	5	9	Boreal Shield_E
7068160	48.45	-71.22	22.9	1979	1991	5	9	Boreal Shield_E
7073652	46.98	-73.18	320	1979	1982	5	9	Boreal Shield_E
708DBCE	47.43	-79.02	274.3	1979	1994	5	9	Boreal Shield_E
7090120	48.57	-78.13	310	1979	1994	5	9	Boreal Shield_E
8501900	53.32	-60.42	48.8	1979	1999	5	9	Boreal Shield_E
4061861	57.35	-107.13	494.6	1979	1993	5	9	Boreal Shield_W
4064150	55.15	-105.27	379.2	1979	1994	5	9	Boreal Shield_W
5061376	53.85	-94.65	235.6	1979	2002	5	9	Boreal Shield_W
5062734	56.78	-98.97	259.1	1979	1988	5	9	Boreal Shield_W

5062922	55.8	-97.86	224.03	1979	1990	5	9	Boreal Shield_W
506B0M7	54	-97.8	217	1979	2000	5	9	Boreal Shield_W
5060600	58.74	-94.07	29.26	1979	2004	5	9	Hudson Plain
6075425	51.27	-80.65	10	1979	1992	5	9	Hudson Plain
6104025	45	-75.63	99.4	1979	1995	5	9	MixedWood Plain
6105976	45.38	-75.72	79.2	1979	1998	5	9	MixedWood Plain
6126552	43.25	-81.85	182.9	1979	1984	5	9	MixedWood Plain
6131982	42.87	-80.55	231.6	1979	1995	5	9	MixedWood Plain
6133360	42.03	-82.9	190.5	1979	1994	5	9	MixedWood Plain
6137147	42.45	-81.88	205.7	1979	1985	5	9	MixedWood Plain
6137730	42.85	-80.27	240.5	1979	1982	5	9	MixedWood Plain
6139538	42.3	-83.07	179.8	1979	1979	5	9	MixedWood Plain
6142285	43.65	-80.42	376.4	1979	1984	5	9	MixedWood Plain
6153300	43.28	-79.88	102.1	1979	1996	5	9	MixedWood Plain
6164433	44.34	-78.74	262.1	1979	1984	5	9	MixedWood Plain
7014160	45.81	-73.43	21	1979	1995	5	9	MixedWood Plain
7016900	46.73	-71.5	57.9	1979	1989	5	9	MixedWood Plain
7025745	45.12	-74.05	45.7	1979	1994	5	9	MixedWood Plain
7026839	45.43	-73.93	39	1979	1992	5	9	MixedWood Plain
7027361	45.57	-72.92	33	1979	1994	5	9	MixedWood Plain
1078209	54.82	-126.16	722	1979	1999	5	9	Montane Cordillera
1123468	50.5	-121	1470.1	1979	1983	5	9	Montane Cordillera
1123970	49.96	-119.38	429.5	1979	1998	5	9	Montane Cordillera
1124980	49.8	-119.2	1249.7	1979	1994	5	9	Montane Cordillera
1125223	49.12	-119.68	1862.3	1979	1979	5	9	Montane Cordillera
1126150	49.46	-119.6	344.42	1979	1992	5	9	Montane Cordillera
1127360	49.35	-120.55	940	1979	1996	5	9	Montane Cordillera
1127800	49.57	-119.65	454.2	1979	1994	5	9	Montane Cordillera
1141457	49.34	-117.77	435	1979	1998	5	9	Montane Cordillera
1142574	50.24	-116.97	548.6	1979	1999	5	9	Montane Cordillera
1153335	49.78	-115.73	774.2	1979	1979	6	9	Montane Cordillera
1160899	52.13	-119.29	690.4	1979	1999	5	9	Montane Cordillera
1175122	52.05	-118.59	579.1	1979	1983	5	9	Montane Cordillera

1183FL0	56.02	-122.2	678.2	1979	1979	5	9	Montane Cordillera
3053600	51.03	-115.03	1391.1	1979	2004	5	9	Montane Cordillera
2403500	74.72	-94.97	67.68	1979	2004	6	9	Northern Arctic
1016940	48.62	-123.42	61	1979	1998	5	9	Pacific Maritime
1021830	49.72	-124.9	25.6	1979	1999	5	9	Pacific Maritime
1031413	48.9	-125	61	1979	1990	5	9	Pacific Maritime
1100120	49.24	-121.76	15	1979	1994	5	9	Pacific Maritime
1108487	49.25	-123.25	76	1979	1990	5	9	Pacific Maritime
3012205	53.32	-113.58	723.3	1979	1985	5	9	Prairie
3012295	53.42	-113.55	693.7	1979	1986	5	9	Prairie
3015311	52.37	-110.25	666.6	1979	1979	7	9	Prairie
3016761	53.48	-112.03	635.8	1979	1994	5	9	Prairie
3023720	52.47	-113.75	847.3	1979	1995	5	9	Prairie
3031093	51.11	-114.02	1084.1	1979	1994	5	9	Prairie
3033890	49.7	-112.77	910	1979	1989	5	9	Prairie
3036652	51.08	-114.13	1112.2	1979	1984	5	9	Prairie
3036681	50.05	-112.13	778.8	1979	1989	5	9	Prairie
3040223	49.25	-110.01	944.9	1979	2003	5	9	Prairie
3044200	49.12	-110.47	934.2	1979	1997	5	9	Prairie
3047365	49.02	-112.95	1310.6	1979	1984	5	9	Prairie
4010879	50.37	-102.57	599.8	1979	1994	5	9	Prairie
4012166	50.38	-104.17	647.7	1979	1980	5	9	Prairie
4012400	49.22	-102.97	580.3	1979	2004	5	9	Prairie
4013490	50.5	-103.68	604.1	1979	1994	5	9	Prairie
4015344	49.88	-103.02	618.7	1979	1986	5	9	Prairie
4015680	49.72	-105.37	685.8	1979	1998	5	9	Prairie
4016560	50.43	-104.67	577.6	1979	1995	5	9	Prairie
4018760	49.65	-103.83	569.7	1979	2007	5	9	Prairie
4019035	51.77	-104.2	560.8	1979	2004	5	9	Prairie
4019080	51.27	-102.47	498.3	1979	2002	5	9	Prairie
4020701	51.08	-106.97	594.4	1979	1980	5	9	Prairie
4028060	50.27	-107.73	825	1979	2006	5	9	Prairie
4031776	49.3	-109.48	941.5	1979	1985	5	9	Prairie

4038400	49.37	-107.85	808	1979	2006	5	9	Prairie
404037Q	51.32	-108.42	637	1979	1986	5	9	Prairie
4043920	51.47	-109.17	683.4	1979	1984	5	9	Prairie
4055736	51.48	-107.05	541	1979	1996	5	9	Prairie
4057180	52.15	-106.6	496.8	1979	1992	5	9	Prairie
5010140	49.28	-99.29	449.9	1979	1987	5	9	Prairie
5021054	49.65	-97.12	234.4	1979	2002	5	9	Prairie
5021848	49.18	-98.08	297.5	1979	1998	5	9	Prairie
5023222	49.92	-97.23	238.7	1979	1996	5	9	Prairie
5023261	49.95	-97.1	232.6	1979	1979	5	9	Prairie
5040764	50.18	-98.38	248.4	1979	1998	5	9	Prairie
5043158	50.71	-99.53	351	1979	1996	5	9	Prairie
2202800	65.28	-126.8	72.54	1979	1984	5	9	Taiga Plain
7095480	53.2	-70.9	536.1	1979	1985	6	9	Taiga Shield_E
8501132	53.55	-64.1	439.5	1979	1992	5	9	Taiga Shield_E
2204100	62.46	-114.44	205.7	1979	1996	5	9	Taiga Shield_W

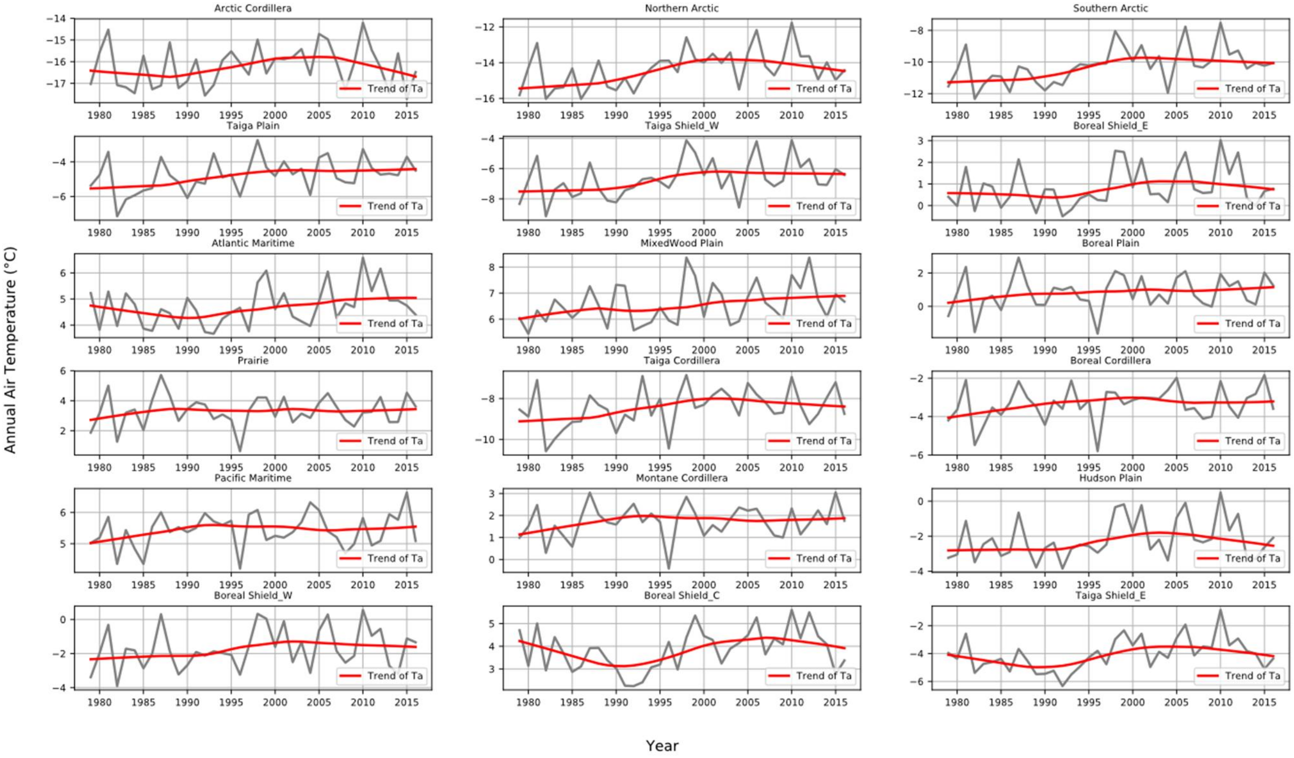

Figure S1. The temporal profile of annual air temperature (°C) and the trends identified by the LOESS regression in 1979–2016 in the 18 ecozones.

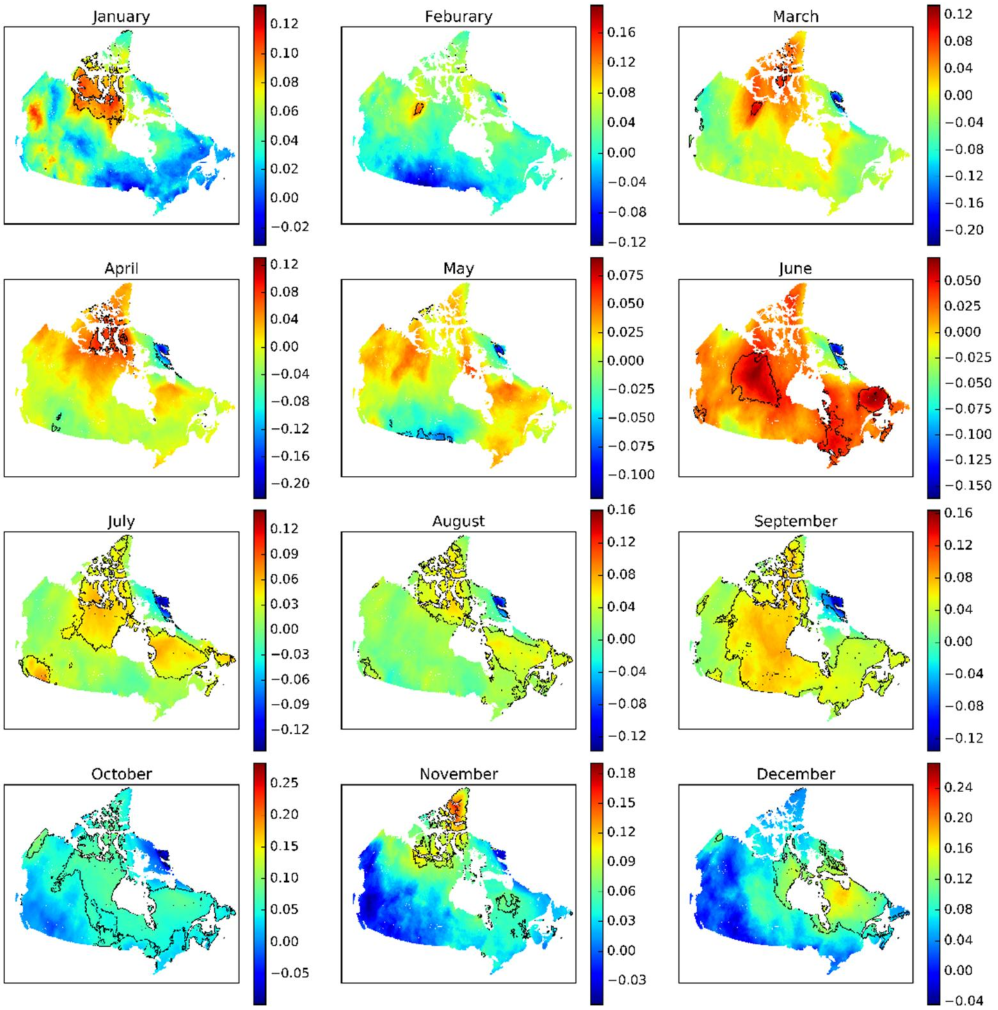

Figure S2. The trends of monthly air temperature (°C/month) during the period of 1979-2016 (the black polygons highlight where the trend is statistically significant at the 95% confidence interval).

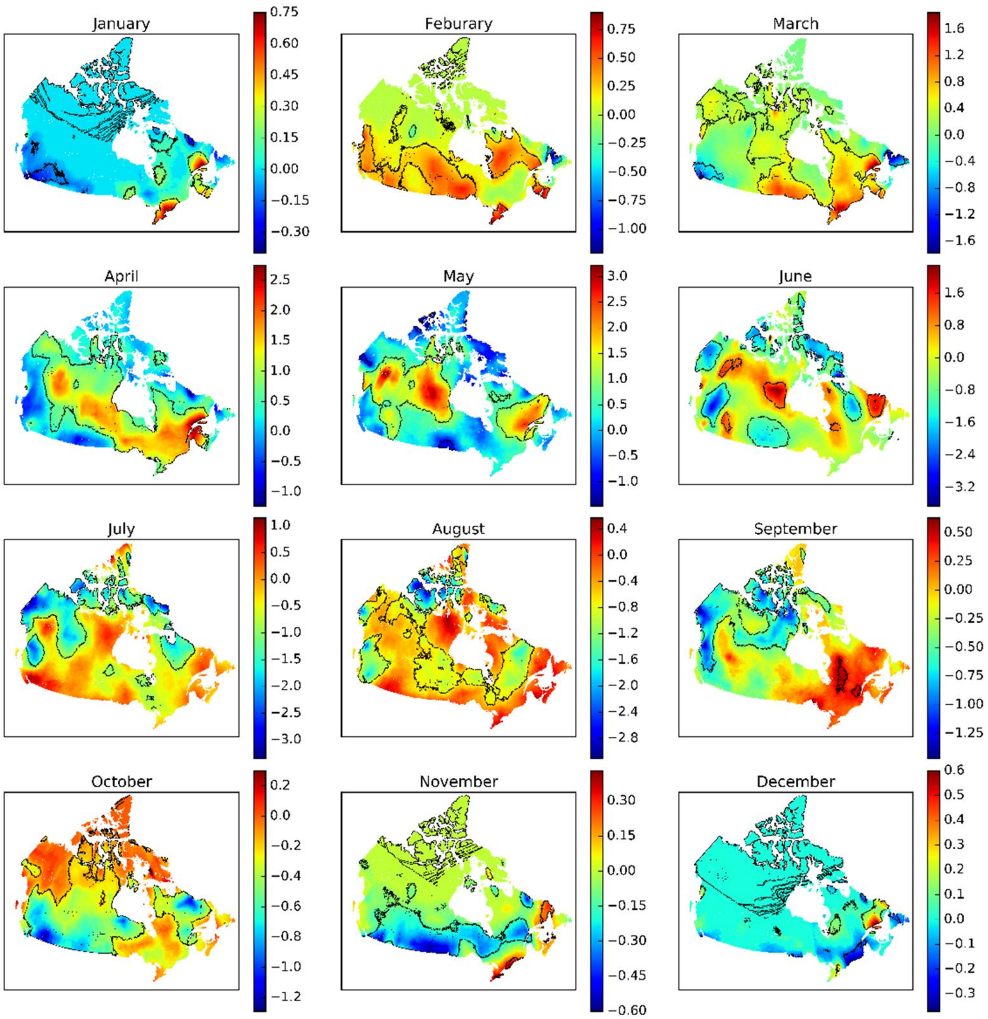

Figure S3. The trends of monthly downward shortwave radiation ($MJ/m^2/month$) during the period of 1979-2016 (the black polygons highlight where the trend is statistically significant at the 95% confidence interval).

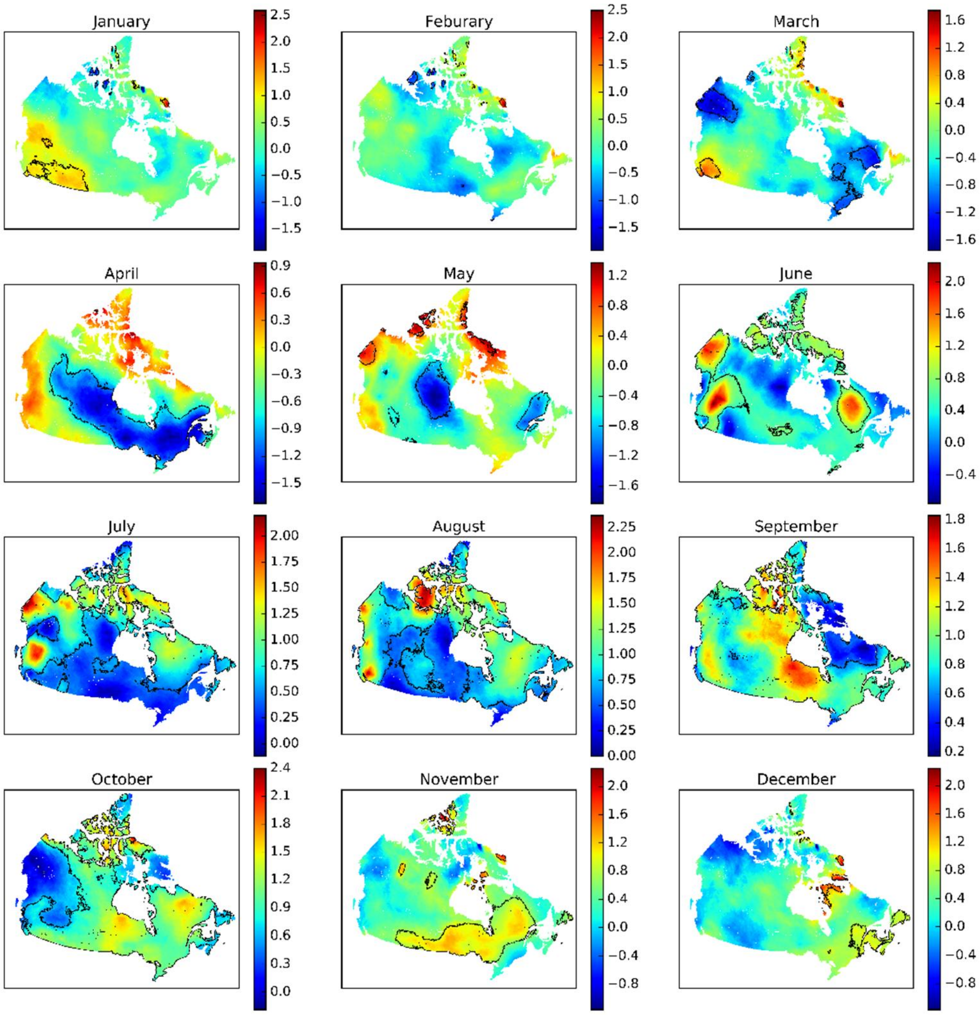

Figure S4. The trends of monthly downward longwave radiation (MJ/m^2/month) during the period of 1979-2016 (the black polygons highlight where the trend is statistically significant at the 95% confidence interval).

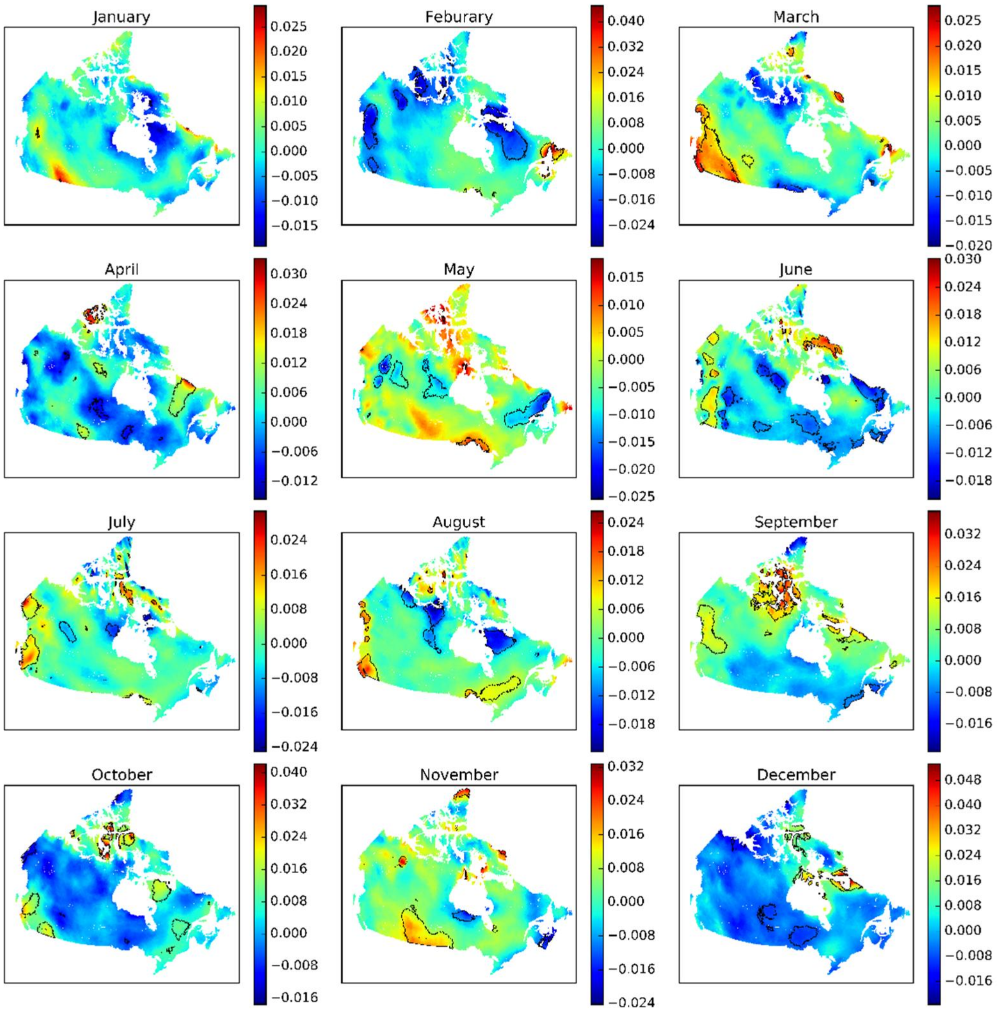

Figure S5. The trends of monthly wind (m/s) during the period of 1979-2016 (the black polygons highlight where the trend is statistically significant at the 95% confidence interval).

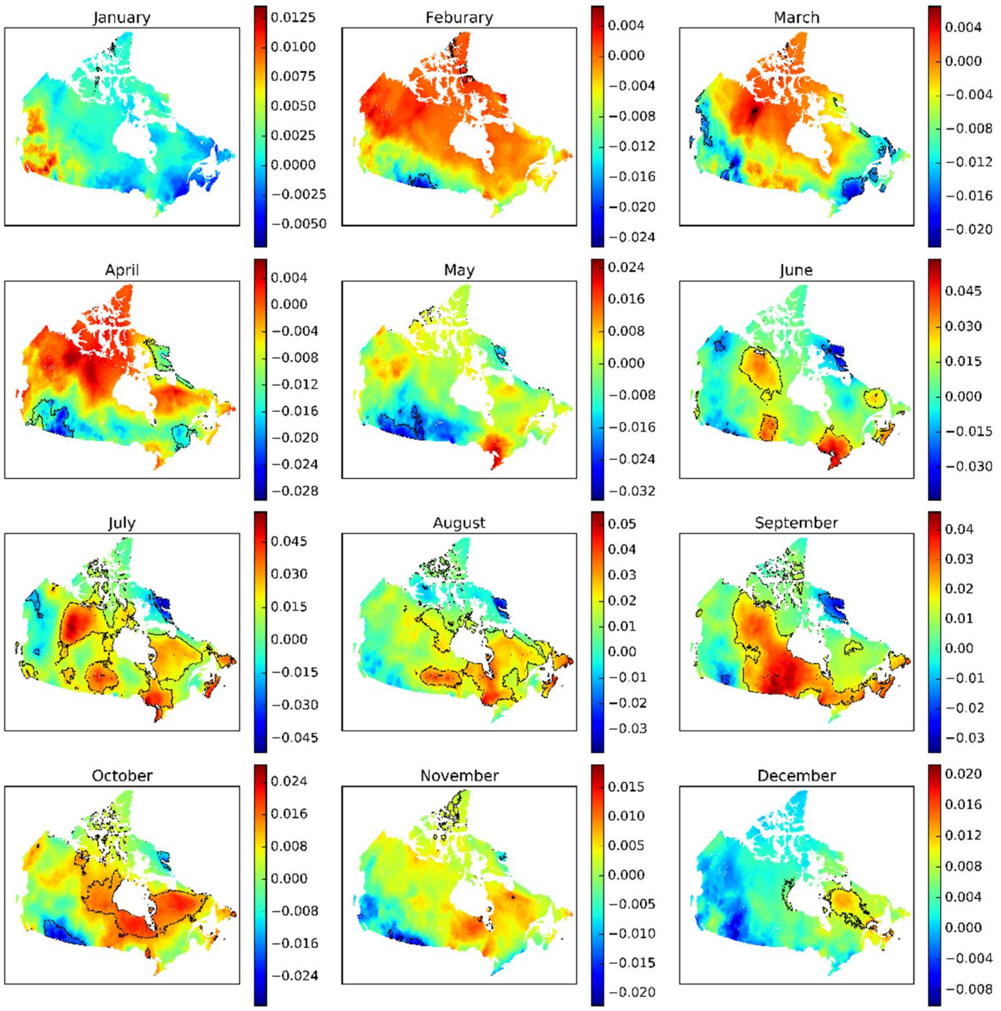

Figure S6. The trends of monthly specific humidity (g H_2O/kg Air/month) during the period of 1979-2016 (the black polygons highlight where the trend is statistically significant at the 95% confidence interval).
